# Population-level norm values by EQ-5D-3L in Hungary - a comparison of survey results from 2022 with those from 2000

**DOI:** 10.1007/s11136-024-03699-9

**Published:** 2024-06-05

**Authors:** András Inotai, Dávid Nagy, Zoltán Kaló, Zoltán Vokó

**Affiliations:** 1 Center for Health Technology Assessment, Semmelweis University, Üllői út 25, Budapest, 1091 Hungary; 2 Syreon Research Institute, Mexikói út 65, Budapest, 1142 Hungary

**Keywords:** Health-related quality of life, Value set, EQ-5D, EuroQoL, Population survey, Adolescent

## Abstract

**Purpose:**

Although population norms of the EQ-5D-3L instrument had been available in Hungary since 2000, their evaluation was based on a United Kingdom (UK) value set. Our objective was to estimate the population norms for EQ-5D-3L by using the new Hungarian value set available since 2020, to extend the scope to adolescents, and to compare with norms from 2000.

**Methods:**

A cross sectional EQ-5D-3L survey representative of the Hungarian population was conducted in 2022. The EQ-5D-3L dimensional responses were analyzed by age and sex and compared with the survey from 2000, by estimating population frequencies with their 95% confidence intervals; index values were evaluated by both value sets.

**Results:**

Altogether, 11,910 respondents, aged 12 or more (578 between 12 and 17), completed the EQ-5D-3L. There was a notable improvement in reporting problems for both sexes (age 35–64) regarding the pain/discomfort and anxiety/depression compared to 2000. Below the age 44, both sexes had an EQ-5D-3L index plateau of 0.98, while above the age 55, men tended to have numerically higher index values compared to women, with the difference increasing with older age. Improvement in dimensional responses were also translated to numerically higher index values for both sexes between ages 18 and 74 compared to 2000. Multivariate regression analysis showed that higher educational attainment, lower age, larger household size, and active occupational status were associated with higher index values.

**Conclusion:**

Over the past 22 years, there was a large improvement in HRQoL of the middle-aged to elderly men and women in Hungary.

**Supplementary Information:**

The online version contains supplementary material available at 10.1007/s11136-024-03699-9.

## Abstract (plain language summary)

Health states can be described by a combination of statements of health-related quality of life measures. ‘Value sets’ are numerical expressions of how preferred a health state is. The provision of population-level health-related quality of life estimates (also known as ‘population norms’) are expected to improve the precision of patient-level clinical decision making, and health economic and public health studies. However, preference towards these health states is influenced by culture, resulting in differences across populations. While responses for the EQ-5D-3L instrument for adults have been available in Hungary since 2000, the evaluation of these responses was based on a ‘value set’ from the United Kingdom, rather than a Hungarian one.

This research, utilizing the newly introduced Hungarian ‘value set’ (available since 2020) for the EQ-5D-3L instrument, offers a larger sample size, inclusion of adolescents and potentially improved sampling compared to the prior research conducted in 2000. Comparison of the two surveys allows us to estimate changes in both dimensional responses and overall health-related quality of life of the population over a 20-year time horizon, while we also compare the impact of different ‘value sets’ on health-related quality of life assessment. A large EQ-5D-3L improvement was observed in middle-aged-to-elderly people.

**Supplementary Information:**

The online version contains supplementary material available at 10.1007/s11136-024-03699-9.

## Introduction

Health-related quality of life (HRQoL) is a subjective multi-dimensional concept that includes dimensions related to physical, mental, emotional, and social functioning and that goes beyond clinical measures of health status [1]. Standardized and validated generic and disease-specific measures are used to estimate the HRQoL of individuals [2]. Generic measures are universally applicable in a wide range of diseases.

One of the most widely used generic HRQoL instrument is the EQ-5D developed by the EuroQoL Group. All EQ-5D questionnaires include a descriptive system focusing on five dimensions: mobility, self-care, usual activities, pain/discomfort and anxiety/depression, and a vertical visual analogue scale for self-assessment of health status (EQ VAS). EQ-5D-3L and −5L are popular generic measures among adults, and EQ-5D-Y-3L and −5L are targeted for the younger population with a child-friendly wording [3]. In the EQ-5D-3L index, each dimension has three levels (3L), with level 1 (L1) denoting no problems and level 3 (L3) denoting ‘extreme problems/unable to/confined to bed’. (In the 5L version, each dimension has five levels). The EQ VAS (also known as EQ-5D Thermometer) is ranged from 0 to 100 (‘the best and worst health you can imagine’) [4, 5].

EQ-5D instruments’ patient-reported values (profile scores) can be converted to an index score using a selected algorithm. Such algorithms are based on surveying the general public’s preferences for different combinations of health states, resulting in ‘value sets’ that are numerical expressions of how preferred a health state is. Therefore, measures such as EQ-5D are also referred to as a ‘preference-based’ or ‘preference-accompanied measure’. The measurement interval of the value set developed originally for EQ-5D-3L by Dolan (using the time trade-off (TTO) method, based on a United Kingdom (UK) population sample) is between -0.594 (health state 33333) to 1 (health state 11111), where the value of 0.0 refers to being dead, 1.0 refers to full health (‘UK value set’) [6–9].

However, as individuals in different cultures or countries may assign different values to certain health states, many countries other than the UK have also developed their own value sets since the introduction of the EQ-5D-3L [10]. Since 2020, a Hungarian value set has also been available for both the EQ-5D-3L and the 5L, based on a non-probability quota sample of 1000 respondents in the Hungarian general population, by using a composite TTO method [11] (‘Hungarian (HU) value set’). Quotas are set by age and sex. Index values in the Hungarian value set range from -0.865 to 1 for the EQ-5D-3L health states. EQ-5D is also among the preferred instruments for health technology assessment (HTA) in Hungary [12].

Population norm values are used as a reference to estimate the HRQoL decrement of a patient population with different diseases. They are applied both in micro-level clinical decision-making (estimating HRQoL) and in health economic models, and macro-level public health decisions (estimating health loss/burden of disease [13]). In Hungary, the poor general health status of the population has led to several population-level health surveys by using the EQ-5D-3L instrument. Most importantly, in 2000, a national health survey was conducted on a representative sample of 5503 individuals representing the whole population, age and sex, by using a paper-based self-administered EQ-5D-3L [14, 15]. This was followed by the European Value of a Quality Adjusted Life Year (EuroVaQ) project in 2010, in which a population sample of 2281 individuals completed a web-based EQ-5D-3L (although it was intended to be representative, the authors reported that the sample overrepresented women and underrepresented the elderly population) [16]. Both surveys applied the UK value set and used an adult sample. The publication of the Hungarian value set in 2020 provided a solid platform for re-estimating the index values of the EQ-5D-3L for the Hungarian adult population. In parallel, the increasing number of HTA submission dossiers with pediatric/adolescent indication also necessitated defining population norm values for the under-18 population to have a more accurate estimate of their cost effectiveness in the Hungarian setting. This led to the need for population health surveys covering an extended age range, also including the 12–17 age band.

This paper aims to present an overview of the HRQoL of the Hungarian population aged 12 years and older based on a representative random sample using the EQ-5D-3L instrument, and to compare the results with the prior survey from 2000.

## Methods

### Survey

This survey was part of a larger cross-sectional national survey on travelling habits of Hungarian people, conducted by the Hungarian Central Statistical Office on a quarterly basis. EQ-5D-3L was an add-on to this survey using standard demographic questions, but without any other health care-related questions.

### Selection of instrument and age

Although the EQ-5D-5L was also considered for the study, the EQ-5D-3L was ultimately selected. This allows for a comparison with prior population surveys [14–16], with several existing disease-specific HRQoL studies using EQ-5D-3L (conducted both at Semmelweis University [e.g. 17–22] and at other research centers in Hungary [e.g. 23–28]), and also with high quality validation studies [29–32]. As the EuroQoL Group recommends the EQ-5D-3L adult version for adolescents aged 16 and above, and considers both the EQ-5D-3L adult version and the EQ-5D-Y-3L to be acceptable for adolescents aged 12–15 years, for logistical simplicity (i.e., using only one instrument) and in concordance with EQ-5D-Y-3L user guide, the minimum eligible age was 12 years in this study, while the EQ-5D-3L version was applied also for adolescents aged 12–17 years. As health economic models benefit from more precise index value data and also to minimize residual confounding by age, index values are reported per a 5-year age band. However, to ensure comparability with the national health survey from 2000, also a 10-year age band was applied in this study.

### Research ethics

The study protocol was approved by the Medical Research Council – Scientific and Ethical Committee in Hungary (number of ethical approval: IV/2292-1 2022/EKU), and research was performed in accordance with the ethical standards of the 1964 Declaration of Helsinki [33].

### Population survey sampling

Primary sampling units (PSUs) were settlements, and the secondary sampling units were dwellings in Hungary. The settlements were stratified by county and size. Larger settlements were selected with certainty. No general national threshold was applied to define certainty PSUs, it varied from county to county. The probability of selecting smaller PSUs was proportional to their size in terms of dwellings. Dwellings were randomly selected within a settlement. All household members aged 12 years or older were included in the survey.

### Population survey weighting

Design weights were calculated based on the sampling design. After the data collection, these were calibrated to correct for nonresponse by geographical region to population size, sex and age distribution, development category of the settlement, and household size, so that we could provide unbiased estimates on the level of the population. These analytical weights were used in the statistical analysis. They had a range of 140–2500, reflecting the number of people a study participant represented. To include 11,910 respondents aged 12 or more, 15,058 individuals were contacted in 7578 households, resulting in a response rate of 79%. Of all the EQ-5D-3L questionnaires, 2.81% were self-administered online, 46.64% by telephone interview, and 50.55% by personal interview, between 1st April and 2nd May 2022. The questionnaire was designed in such a way that it did not allow item nonresponse, ‘I do not know/I do not respond’ answers. As the questionnaire was short, withdrawing the participation in the meantime did not happen. No deletion of responders due to lack of data or imputation was necessary. The participant (unit)-level non-response was corrected for in the weighting, the description of which was provided earlier.

### Statistical analysis methods

We used the survey module of the statistical software STATA 16.1 [34]. Dimensional distribution analysis was performed by estimating population frequencies with their 95% confidence intervals, by using the same age bands and reporting structure as applied in the 2000 national health survey. Mean EQ-5D-3L index values with their 95% confidence intervals were estimated for the target population by age and sex using “svy: mean” procedure with an analytically derived variance estimator associated with the sample mean. Weighted multiple linear regression analyses were applied by using sex, age (adults only), education, occupation, and household size as explanatory variables applying “svy: regress” procedure. Additionally, we fitted a regression model with the interaction terms between sex and age bands adjusted for education, occupation, and household size. Design-based standard errors were estimated taking into account the stratified cluster sampling.

## Results

### Sample characteristics

Table 1 describes the baseline characteristics of the sample including age, sex, geographical region, education, occupation and household size, also by the mode of administration. In the unweighted sample 54.3% of participants were women, 33.8% were students and 11.5% participants were from Budapest. Online responders tend to be younger and living in Budapest with a higher educational attainment.

**Table 1:** General characteristics of study participants in 2022 population survey

Characteristics	Total		Personal interview		Telephone interview		Self-administered online	
N, %	11,910	100	6020	100	5555	100	335	100
*Sex*								
Women	6466	54.3	3296	54.7	2998	54.0	172	51.3
Men	5444	45.7	2724	45.3	2557	46.0	163	48.7
*Age (year)*								
12–17	578	4.9	306	5.1	252	4.5	20	6.0
18–24	818	6.9	405	6.7	393	7.1	20	6.0
25–34	1456	12.2	745	12.4	665	12.0	46	13.7
35–44	1664	14.0	852	14.1	752	13.5	60	17.9
45–54	1945	16.3	943	15.7	933	16.8	69	20.6
55–64	1892	15.9	956	15.9	883	15.9	53	15.8
65–74	2176	18.3	1081	18.0	1050	18.9	45	13.4
75–84	1105	9.3	576	9.6	509	9.2	20	6.0
85+	276	2.3	156	2.6	118	2.1	2	0.6
*Geographical region (residence)*								
Budapest (Central Hungary)	1364	11.5	487	8.1	771	13.9	106	31.6
Pest (Central Hungary)	1005	8.4	384	6.4	570	10.3	51	15.2
Central Transdanubia	1433	12.0	792	13.1	627	11.3	14	4.2
Western Transdanubia	1280	10.8	621	10.3	634	11.4	25	7.5
Southern Transdanubia	1310	11.0	645	10.7	627	11.3	38	11.3
Northern Hungary	1557	13.1	974	16.2	548	9.9	35	10.4
Northern Great Plain	2153	18.1	1112	18.5	1002	18.0	39	11.6
Southern Great Plain	1808	15.2	1005	16.7	776	14.0	27	8.1
Education (highest level completed)*								
Less than 8 years in primary school	438	3.7	257	4.3	174	3.2	7	2.2
Primary school	2481	21.0	1432	23.9	1017	18.4	32	9.9
Secondary school	6447	54.5	3251	54.3	3071	55.6	125	38.7
University	2469	20.9	1049	17.5	1261	22.8	159	49.2
Occupation**								
Employed/self-employed	5907	49.6	2947	49.0	2763	49.8	197	58.8
Unemployed	247	2.1	142	2.4	100	1.8	5	1.5
Pensioner	999	8.4	465	7.7	496	8.9	38	11.3
Student	4023	33.8	2063	34.3	1880	33.9	80	23.9
Other inactive***	723	6.1	396	6.6	312	5.6	15	4.4
*Household size*								
1	2180	18.3	1175	19.5	957	17.2	48	14.3
2	4083	34.3	2088	34.7	1886	34.0	109	32.5
3	2349	19.7	1121	18.6	1137	20.5	91	27.2
4	1916	16.1	946	15.7	904	16.3	66	19.7
5	950	8.0	478	7.9	455	8.2	17	5.1
6	267	2.2	123	2.0	140	2.5	4	1.2
7–9	165	1.4	89	1.5	76	1.3	0	0

*75 out of 11,910 did not answer this question

**11 out of 11,910 did not answer this question

***unable to work, entitled to child benefit, householder, not working for other reason

### EQ-5D-3L dimensional, index (using HU value set), and VAS norms by age and sex

Supplementary Table 1 reports weighted EQ-5D-3L questionnaire responses by mode of administration. Problems (L2 + L3) in anxiety/depression were reported slightly more frequently in the case of online self-administration, compared to the telephone and online interviews. Supplementary Table 2 reports the weighted EQ-5D-3L dimensional responses for 5- and 10-year age bands. Among the five dimensions, anxiety/depression was the one where both sexes reported problems (L2 + L3) even in the younger age bands. Towards higher age bands, mobility, pain/discomfort and usual activities were increasingly associated with problems. Similarly, L3 impairments were reported mainly in the dimensions of usual activities and pain/discomfort by older adults. Comparing the two sexes, younger men tended to report slightly more problems in all dimensions; on contrary, above 75 women tended to report more problems. Along with older ages, women tended to report more L3 impairments in the dimension of pain/discomfort, while men tended to report more L3 impairments in self-care, compared to the other sex.

Table 2 reports the weighted EQ-5D-3L index values from the 2022 population survey (evaluated by using the Hungarian value set) of the 12-year-old and older by 5- and 10-year age bands. Using the Hungarian value set, index values showed a plateau of 0.98 under age of 45. Men older than 54 years generally had numerically higher, although statistically not significantly different index values compared to women, with the difference slightly increasing with older ages. In contrast to adults, the index values for girls were minimally higher than for boys among participants under 18 years of age.

**Table 2:** Weighted EQ-5D-3L index values (using the Hungarian value set (by Rencz et al.)) and EQ VAS scores per 5- and 10-year age bands, by age and sex in 2022 population survey

	Index values (Hungarian value set) (0–1)*		EQ VAS (0-100)**	
	Mean	95% CI	Mean	95% CI
Adolescents				
men 12–15 years	0.987	(0.967; 1.007)	95.8	(94.6; 97.0)
men 16–17 years	0.990	(0.982; 0.999)	94.8	(92.5; 97.1)
Adults				
men 18–24 years	0.982	(0.971; 0.993)	92.4	(91.3; 93.6)
Adults by 10-yr age band				
men 25–34 years	0.979	(0.972; 0.987)	89.4	(88.2; 90.5)
men 35–44 years	0.984	(0.980; 0.989)	89.0	(88.0; 90.0)
men 45–54 years	0.962	(0.953; 0.971)	84.2	(83.1; 85.4)
men 55–64 years	0.935	(0.924; 0.946)	78.9	(77.4; 80.3)
men 65–74 years	0.885	(0.869; 0.901)	71.9	(70.3; 73.4)
men 75–84 years	0.792	(0.761; 0.824)	63.2	(60.7; 65.6)
men 85 + years	0.666	(0.562; 0.771)	56.8	(52.2; 61.5)
Adults by 5-yr age band				
men 25–29 years	0.983	(0.976; 0.989)	90.1	(88.6; 91.7)
men 30–34 years	0.976	(0.963; 0.990)	88.7	(87.2; 90.2)
men 35–39 years	0.986	(0.979; 0.992)	89.4	(88.0; 90.7)
men 40–44 years	0.983	(0.978; 0.989)	88.7	(87.4; 90.0)
men 45–49 years	0.969	(0.958; 0.979)	85.4	(83.9; 86.9)
men 50–54 years	0.954	(0.938; 0.970)	82.8	(81.3; 84.3)
men 55–59 years	0.946	(0.932; 0.959)	80.6	(78.6; 82.5)
men 60–64 years	0.924	(0.909; 0.939)	77.1	(75.3; 78.9)
men 65–69 years	0.886	(0.865; 0.908)	73.5	(71.6; 75.4)
men 70–74 years	0.883	(0.860; 0.906)	69.6	(67.6; 71.7)
men 75–79 years	0.810	(0.768; 0.853)	66.4	(63.4; 69.4)
men 80–84 years	0.763	(0.712; 0.813)	57.8	(54.4; 61.2)
Adolescents				
women 12–15 years	0.991	(0.983; 0.999)	95.5	(94.0; 97.0)
women 16–17 years	0.994	(0.988; 1.001)	96.1	(94.9; 97.3)
Adults				
women 18–24 years	0.973	(0.951; 0.995)	92.4	(91.1; 93.7)
Adults by 10-yr age band				
women 25–34 years	0.983	(0.977; 0.989)	90.4	(89.4; 91.4)
women 35–44 years	0.978	(0.971; 0.985)	87.8	(86.7; 89.0)
women 45–54 years	0.960	(0.952; 0.967)	83.9	(82.8; 85.0)
women 55–64 years	0.926	(0.915; 0.936)	77.3	(76.0; 78.5)
women 65–74 years	0.870	(0.858; 0.883)	70.7	(69.4; 71.9)
women 75–84 years	0.772	(0.743; 0.800)	62.7	(60.7; 64.6)
women 85 + years	0.638	(0.573; 0.703)	55.5	(52.3; 58.7)
Adults by 5-yr age band				
women 25–29 years	0.987	(0.982; 0.993)	91.3	(90.1; 92.5)
women 30–34 years	0.979	(0.968; 0.989)	89.5	(87.9; 91.0)
women 35–39 years	0.980	(0.971; 0.990)	87.5	(85.7; 89.3)
women 40–44 years	0.976	(0.967; 0.986)	88.1	(86.8; 89.4)
women 45–49 years	0.972	(0.964; 0.979)	86.2	(84.8; 87.6)
women 50–54 years	0.947	(0.933; 0.961)	81.4	(79.7; 83.1)
women 55–59 years	0.938	(0.923; 0.953)	79.0	(77.3; 80.8)
women 60–64 years	0.914	(0.900; 0.927)	75.6	(74.1; 77.0)
women 65–69 years	0.893	(0.879; 0.907)	73.6	(72.1; 75.1)
women 70–74 years	0.842	(0.822; 0.862)	67.1	(65.3; 68.9)
women 75–79 years	0.802	(0.777; 0.828)	64.0	(61.7; 66.4)
women 80–84 years	0.726	(0.673; 0.778)	60.6	(58.0; 63.3)

yr: year, VAS: visual analog scale; CI: confidence interval

*reference points: 0 for death, 1 for full health

**measurement interval

Table 2 also reports EQ VAS data in a similar age structure. Above the age of 34, men tended to have minimally higher EQ VAS compared to women in every 10-year age bands. Above age 39, EQ VAS tended to decrease in every consecutive 5-year age band in both sexes.

### Comparison of the dimensional responses between 2000 and 2022

Supplementary Table 3 reports comparison of problems by dimension between 2000 and 2022, using the data structure of the 2000 national survey. There was a notable improvement in reporting problems (L2 + L3) for both sexes in the age band of 35–64 in all dimensions except for self-care, with a major improvement in both pain/discomfort and anxiety/depression, especially for women. Some improvement was also seen in pain/discomfort (for age 18–34) and anxiety/depression (for both age 18–34 and 65+) from 2000 to 2022 for both sexes, but a numerically larger one for women.

### Comparison of index values (using the UK value set) between 2022 and 2000

**Fig. 1:**
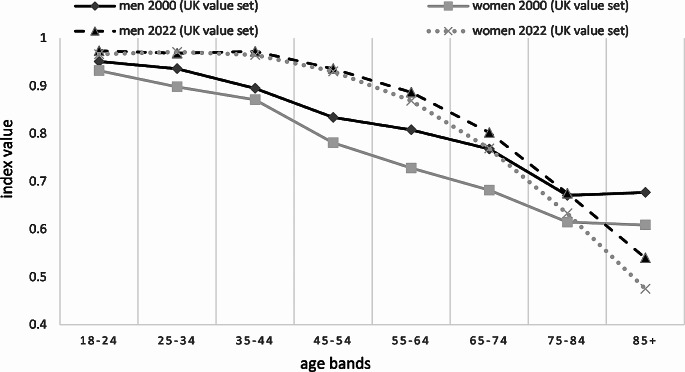
Comparison of EQ-5D-3L index values (using the UK value set) in both the 2022 population survey and the 2000 national health survey

Figure 1 shows a comparison of weighted EQ-5D-3L index values of the 2022 population survey with the 2000 national health survey for 10-year age bands, both using the UK value set to ensure consistency. Supplementary Table 4 reports the weighted EQ-5D-3L index values for the same comparison for 10-year age bands. The 2022 population survey resulted in higher index values than the 2000 national health survey between age 18–74, especially between the ages 35 and 64, where better dimensional responses of the 2022 population survey were also translated into higher weighted index values for both sexes compared to the 2000 national health survey. On contrary, for the 85+ age band, the 2022 population survey showed markedly lower weighted index values compared to the 2000 national health survey. In that study, the difference by sex was numerically even larger in all relevant age bands.

### Comparison of index values derived by the HU and UK value sets for the 2022 population survey

**Fig. 2:**
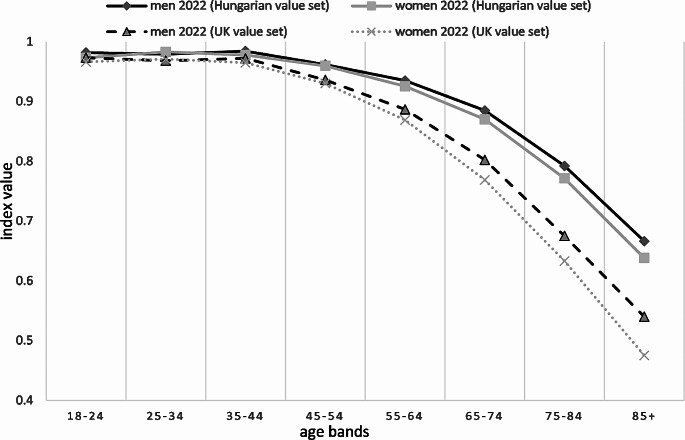
EQ-5D-3L index values (using the UK and Hungarian value set) per 10-year age bands and sex in 2022 population survey

As an overview, Fig. 2 presents the weighted EQ-5D-3L index data for both sexes from the 2022 population survey by using both the Hungarian (default) and the UK value sets for 10-year age bands. Index values derived by the UK value set from the 2022 population survey (see Supplementary Table 4) showed a similar trend to those indices derived by the HU value set from 2022 (see Table 2), but with lower weighted index values above age of 45 and a larger, statistically not significant difference between men and women, especially in older age groups.

### Comparison of VAS results

Supplementary Table 5 reports the weighted EQ VAS numbers for the same comparison for 10-year age bands. EQ VAS numbers were consistently higher in both sexes in 2022 compared to 2000 for with the smallest difference for those aged 85+.

### Regression results

Finally, Table 3 presents the results of the weighted multiple linear regression analysis estimating the EQ-5D-3L index values for adults. Multivariate analysis showed that, after controlling for other variables, sex had no significant impact on the index values, but there was a significant interaction between sex and age (adjusted Wald-test *p*-value: 0.014). In terms of age, the index values significantly differ from the index value of the reference age band (18–24 years) above the age of 49 years. Higher educational attainment was associated with higher index values. Economic activity was used as a nominal variable in the model and showed a significant impact on the index values: employed respondents and students had significantly higher index values than those who were unemployed, retired or inactive for other reasons, the latter having the lowest index values. Finally, a larger household size (a larger number of people living in a household) was also associated with higher index values.

**Table 3:** Association of EQ-5D-3L index values (using the Hungarian value set (by Rencz et al.)) with socio-economic characteristics in adults based on weighted multiple linear regression analysis in 2022 population survey

		Mean difference	95% CI	*p*-value
Sex (reference: men)	women	0.003	(-0.003; 0.009)	0.29
Age (reference: 18-24-year-old)	25–29 years	0.008	(-0.009; 0.025)	0.37
	30–34 years	0.002	(-0.015; 0.019)	0.83
	35–39 years	0.001	(-0.017; 0.018)	0.91
	40–44 years	-0.004	(-0.022; 0.014)	0.66
	45–49 years	-0.012	(-0.030; 0.006)	0.19
	50–54 years	-0.027	(-0.048; -0.005)	0.015
	55–59 years	-0.032	(-0.052; -0.011)	0.003
	60–64 years	-0.032	(-0.053; -0.011)	0.002
	65–69 years	-0.040	(-0.063; -0.018)	0.001
	70–74 years	-0.070	(-0.096; -0.043)	< 0.001
	75–79 years	-0.118	(-0.149; -0.087)	< 0.001
	80–84 years	-0.179	(-0.228; -0.130)	< 0.001
	85 + years	-0.264	(-0.326; -0.201)	< 0.001
Education (reference: less than 8 years in primary school)	Primary school	0.138	(0.044; 0.233)	0.004
	Secondary school	0.172	(0.077; 0.267)	< 0.001
	University	0.193	(0.098; 0.287)	< 0.001
Occupation (reference: employed)	Unemployed	-0.045	(-0.078; -0.012)	0.007
	Student	0.011	(-0.004; 0.026)	0.16
	Pensioner	-0.051	(-0.067; -0.036)	< 0.001
	Other inactive*	-0.083	(-0.107; -0.060)	< 0.001
Household size (reference: 1 person)	2 persons	0.017	(0.007; 0.027)	0.001
	3 persons	0.008	(-0.005; 0.021)	0.22
	4 persons	0.020	(0.008; 0.032)	0.001
	5 persons	0.028	(0.014; 0.042)	< 0.001
	6 persons	0.043	(0.021; 0.065)	< 0.001
	7 persons	0.014	(-0.052; 0.080)	0.69
	8 persons	0.022	(-0.029; 0.073)	0.40
	9 persons	0.019	(-0.015; 0.053)	0.27

*unable to work, entitled to child benefit, householder, not working for other reason

CI: confidence interval

## Discussion

Our results showed that in 2022 among the five dimensions, anxiety/depression was the one where both sexes reported problems in younger age bands; towards higher age bands, mobility, pain/discomfort and usual activities were increasingly associated with problems. Both EQ-5D-3L index values and EQ VAS showed reduction along with age above the age of 44 in both sexes, with men having somewhat higher values compared to women. EQ VAS numbers were consistently higher in 2022 compared to 2000 for both sexes, and there was a large improvement in EQ-5D-3L index values between age 35–64. According to the multivariable analysis, younger age, higher education, being active, and larger household size are associated with better HRQoL.

### Comparison with the national health survey 2000

Over the past 22 years the HRQoL of women aged 25–74 and men aged 35–64 improved considerably. Interestingly, our study could not replicate the relatively high index values observed in the 2000 national health survey for the age 85+ (for both men and women). However, in that survey, only 1% of respondents had this age which meant that some of the outliers may have had a potentially larger impact. Since 2000, the health status of elderly people has improved (life expectancy at age 65 improved from 17.3 to 18.7 for women and 13.4 to 14.6 for men between 2004 and 2016) [35], which (if better HRQoL is also assumed) may contradict our results showing lower index values. It seems that the elderly population with a better HRQoL may have been overrepresented in the 2000 national health survey, and the small number of participants of this age provided less robust estimates for this age band.

Overall, beyond using a more relevant value set for EQ-5D-3L, our study also offers larger sample size, thus enhanced statistical power, narrow age bands for more precise economic evaluations, inclusion of adolescents and potentially improved sampling, compared to the 2000 national health survey. We strongly believe that these factors contribute to more credible population norm estimates.

### Impact of UK and HU value sets on index values from the 2022 population survey

Higher index values derived using the Hungarian value set compared to the UK value set (shown in Fig. 2) can be explained by two key factors. Firstly, the Hungarian value set uses a 0.020 constant (decrement, to be used for health states other than 11111) instead of 0.081 used by UK value set. Secondly and more importantly, the new Hungarian value set does not apply the constant N3 (an additional  -0.269 decrement for L3 responses in any dimension used by UK value set, beyond the respective dimension-specific L3 decrement), as its impact has been considered in larger L3 decrements in the Hungarian value set compared to the UK one. On the other hand, for L2 responses which were reported much more frequently (Supplementary Table 2), the new Hungarian value set tend use smaller decrements compared to the UK one.

### Comparison with other population norms in the region

Nikl et al. published population norms for Hungarian population on a sample of 2000 adults, reported to be broadly representative in terms of sex, age groups, highest level of education, geographical region, and settlement type, using the 15D instrument [36]. The mean 15D index value was 0.810 using the Norwegian 15D value set. In that study, with advancing age categories, the 15D index values showed an inverse U-shaped curve with highest index values of 0.82 for both age bands of 25–34 and 45–54; and numerical results could be considered somewhat consistent with index values (derived by the UK value set) of this research. However, different HRQoL instruments, sample sizes, value sets, recruitment (i.e., voluntary registration from online panel) make the more detailed comparison of the two studies difficult. In Poland, Golicki and Niewada published population norms on a sample of 3963 adults, representative of the Polish population in terms of age, sex, geographical region, education, and socio-professional group, by using a self-administered EQ-5D-5L instrument [37]. To calculate index values, an interim EQ-5D-5L value set for Poland was used based on a crosswalk methodology. Index values (0.96 for those aged 18–24, 0.94 for 35–44, 0.9 for 45–54 and 0.81 for 65–74, respectively) were broadly consistent yet somewhat lower than the Hungarian ones (derived by the Hungarian value set). Again, differences in the applied instruments, sample sizes and administration make further direct comparison of the norms between the two countries difficult. In both studies, similarly to our results, men tend to have higher values in almost all age bands, especially above 35 years. Finally, Zrubka et al. compared EQ-5D-3L population studies from Hungary, Slovenia and Poland and reported issues in terms of comparability due different national characteristics, different data collection methodologies and times [38]. Importantly, data gaps for age 65+ were reported to be a general concern, confirming our findings in the oldest age category in the 2000 national health survey.

### Regression analyses: younger age, higher education, being active and having a larger household size were associated with better HRQoL

To minimize the impact of adult value set applied also for adolescents, our regression analysis included adults only. The lower EQ-5D-3L index in elderly individuals may be explained by the fact that elderly people tend to suffer from more diseases, including multi-morbid conditions [39], which potentially have a major impact on HRQoL. Better education is shown to be associated with healthier lifestyles [40–42], higher participation in prevention programs, and appreciation of being healthy in general. Active occupation (i.e., employee, student) may lead to more physical activities and/or social contact, which may contribute to higher index values. On the contrary, inactive people may lack these, while those involved in childcare may feel isolated, sleepless or experience maternal depression, potentially associated with poorer HRQoL. Finally, interpersonal relationships are likely to be stronger in households with two or more people. Moreover, in larger families, the average household index value seems to be increasing significantly with household size even after controlling for the other factors listed in Table 3. Our findings on the association of age and education with HRQoL were also confirmed by similar conclusions from the 2000 national health survey [14] and the EuroVaQ study [16]. Interestingly, these prior studies also found that sex had a significant impact on the EQ-5D-3L index, as did household income (however, this latter variable was not included in the 2022 population survey). This is in line with our observation that there was an interaction between sex and age in our study.

### Implications and future research

This research has significant policy implications. The new population norm values for those under 18 introduced by this research will have a significant impact both on the accuracy of health burden estimates and the economic evaluation of health technologies for adolescents. Similarly, the application of the new Hungarian value set that truly reflects the preferences of Hungarian people for different health states are expected to improve the accuracy of health burden estimates and economic evaluations for adults. Moreover, as a Hungarian value set for the EQ-5D-Y-3L has also been available since 2022 [43], future research could compare the impact of using the EQ-3D-3L (with the adult value set) and the future use of the EQ-5D-Y-3L (with the new value set for adolescents) to conclude on the applicability of the adult EQ-5D-3L for adolescents aged 12–15 years, as considered to be acceptable by the EuroQoL Group [44]. Finally, this study together with the research conducted in 2000, with some limitations (difference in sampling methodology, mixing EQ-5D-3L administration methods, various factors influencing population change over 20 years etc.), allows researchers to estimate changes in HRQoL of the population over a 20-year time horizon.

### Strengths and limitations

The large representative random sample and the wide age range are the main strengths of this study. Compared to some other large-scale surveys, our study had more robust outreach for those above 65, a cohort especially relevant from public health and health economic point-of-view. However, it has some limitations, too. First, as the intention with the population-level health survey was to establish norm values for previous and ongoing disease-specific research in Hungary, and also to ensure comparability with the 2000 national health survey, the EQ-5D-5L was not considered for this research. Second, different administration methods of the questionnaire may have introduced bias even in a homogeneous sample. Third, the analytical weights had a relatively large range and, as noted by Potter and Zeng [45], ‘extreme variation in the sampling weights can result in excessively large sampling variances when the data and the selection probabilities are not positively correlated’. Finally, using the adult EQ-5D-3L also for 12-15-year-old adolescents may also have had an impact on the results.

## Conclusion

Over the past 22 years, there was a large improvement in reporting problems for both sexes (especially for women) in age 35–64 in EQ-5D-3L dimensions of pain/discomfort and anxiety/depression, compared to 2000. This was also translated to considerably higher index values for middle-aged women and men. Younger age, higher education, being active, and larger household size are associated with better HRQoL. The study, using the new national value set and extended age, is expected to improve the accuracy of economic evaluations and disease burden studies in Hungary.

## Electronic supplementary material

Below is the link to the electronic supplementary material.

Supplementary Material

## Data Availability

All data generated or analyzed during this study are available from the authors upon reasonable request and with permission of the Hungarian Central Statistical Office.
