# Supplementary material for: Population-level norm values by EQ-5D-3L in Hungary - a comparison of survey results from 2022 with those from 2000
Source: Qual Life Res. 2024 Jun 5;33(9):2417–28. doi: 10.1007/s11136-024-03699-9 (PMC11390784; doi:10.1007/s11136-024-03699-9)
Supplement: Supplementary file 1 — Supplementary Material [file 11136_2024_3699_MOESM1_ESM.docx]

Supplementary table 1. Weighted EQ-5D-3L dimensional responses by mode of administration in 2022 population survey

|  |  | Personal interview | | Telephone interview | | Self administered online | |
| --- | --- | --- | --- | --- | --- | --- | --- |
|  | N | 6020 | | 5555 | | 335 | |
|  |  | proportion | sd | proportion | sd | proportion | sd |
| mobility | L1 | 0.788 | 0.227 | 0.824 | 0.197 | 0.815 | 0.203 |
|  | L2 | 0.199 | 0.208 | 0.167 | 0.183 | 0.175 | 0.187 |
|  | L3 | 0.013 | 0.022 | 0.009 | 0.015 | 0.010 | 0.017 |
| self-care | L1 | 0.928 | 0.089 | 0.939 | 0.075 | 0.977 | 0.031 |
|  | L2 | 0.060 | 0.073 | 0.051 | 0.062 | 0.019 | 0.026 |
|  | L3 | 0.011 | 0.016 | 0.009 | 0.013 | 0.003 | 0.005 |
| usual activities | L1 | 0.855 | 0.157 | 0.877 | 0.135 | 0.900 | 0.115 |
|  | L2 | 0.126 | 0.131 | 0.107 | 0.115 | 0.088 | 0.098 |
|  | L3 | 0.019 | 0.027 | 0.015 | 0.021 | 0.012 | 0.016 |
| pain/discomfort | L1 | 0.717 | 0.239 | 0.752 | 0.217 | 0.708 | 0.228 |
|  | L2 | 0.250 | 0.199 | 0.222 | 0.186 | 0.258 | 0.189 |
|  | L3 | 0.033 | 0.044 | 0.026 | 0.034 | 0.033 | 0.043 |
| anxiety/depression | L1 | 0.831 | 0.119 | 0.852 | 0.105 | 0.788 | 0.128 |
|  | L2 | 0.149 | 0.101 | 0.130 | 0.090 | 0.184 | 0.107 |
|  | L3 | 0.021 | 0.018 | 0.017 | 0.014 | 0.027 | 0.210 |

L1 denoting no problems, L2 denoting some/moderate problems and L3 denoting extreme problems/unable to/confined to bed

Supplementary table 2. Weighted EQ-5D-3L dimensional responses per 5- and 10-year age bands by age and sex in 2022 population survey expressed in % of responses within each dimension.

| EQ-5D-3L dimension | mobility | | | self-care | | | usual activities | | | pain/discomfort | | | anxiety/depression | | |
| --- | --- | --- | --- | --- | --- | --- | --- | --- | --- | --- | --- | --- | --- | --- | --- |
| level | L1 | L2 | L3 | L1 | L2 | L3 | L1 | L2 | L3 | L1 | L2 | L3 | L1 | L2 | L3 |
| Adolescents |  |  |  |  |  |  |  |  |  |  |  |  |  |  |  |
| men 12-15 years | 98.3 | 1.2 | 0.6 | 98.6 | 0.8 | 0.6 | 97.8 | 1.6 | 0.6 | 98.6 | 0.8 | 0.6 | 99.2 | 0 | 0.8 |
| men 16-17 years | 99.2 | 0.8 | 0.0 | 97.9 | 2.1 | 0 | 97.9 | 2.1 | 0.0 | 99.2 | 0.8 | 0.0 | 92.6 | 7.4 | 0 |
| Adults |  |  |  |  |  |  |  |  |  |  |  |  |  |  |  |
| men 18-24 years | 97.7 | 2.3 | 0.0 | 98.3 | 1.0 | 0.7 | 96.9 | 2.1 | 1.0 | 96.8 | 2.6 | 0.6 | 93.5 | 6.0 | 0.5 |
| Adults by 10-yr age band |  |  |  |  |  |  |  |  |  |  |  |  |  |  |  |
| men 25-34 years | 98.4 | 1.2 | 0.4 | 99.0 | 0.7 | 0.3 | 98.3 | 1.0 | 0.7 | 94.1 | 5.4 | 0.5 | 94.2 | 4.9 | 0.9 |
| men 35-44 years | 96.8 | 3.2 | 0.0 | 99.8 | 0.1 | 0.1 | 98.6 | 1.3 | 0.1 | 92.7 | 7.1 | 0.2 | 94.2 | 5.5 | 0.3 |
| men 45-54 years | 92.9 | 6.7 | 0.4 | 98.2 | 1.6 | 0.2 | 94.6 | 4.8 | 0.6 | 86.0 | 12.2 | 1.8 | 90.2 | 8.5 | 1.3 |
| men 55-64 years | 83.1 | 16.4 | 0.5 | 96.6 | 2.6 | 0.8 | 88.6 | 10.1 | 1.3 | 71.7 | 26.4 | 1.9 | 85.1 | 13.8 | 1.1 |
| men 65-74 years | 63.1 | 35.3 | 1.6 | 90.3 | 8.4 | 1.3 | 79.9 | 17.4 | 2.7 | 55.0 | 41.0 | 4.0 | 80.2 | 17.8 | 2.0 |
| men 75-84 years | 41.5 | 54.6 | 3.9 | 75.4 | 20.4 | 4.2 | 56.8 | 36.2 | 7.0 | 34.3 | 57.8 | 7.9 | 68.3 | 28.3 | 3.4 |
| men 85+ years | 24.3 | 67.2 | 8.5 | 55.6 | 32.7 | 11.7 | 36.5 | 50.0 | 13.5 | 25.9 | 62.2 | 11.9 | 51.3 | 40.5 | 8.2 |
| Adults by 5-yr age band |  |  |  |  |  |  |  |  |  |  |  |  |  |  |  |
| men 25-29 years | 98.7 | 1.3 | 0 | 99.0 | 1.0 | 0 | 98.8 | 0.5 | 0.7 | 94.1 | 5.4 | 0.5 | 93.4 | 5.8 | 0.8 |
| men 30-34 years | 98.0 | 1.2 | 0.8 | 98.9 | 0.4 | 0.7 | 97.8 | 1.5 | 0.7 | 94.1 | 5.4 | 0.5 | 95.1 | 4.0 | 0.9 |
| men 35-39 years | 97.5 | 2.5 | 0 | 99.6 | 0.2 | 0.2 | 98.9 | 0.9 | 0.2 | 92.9 | 7.1 | 0 | 94.4 | 5.6 | 0.0 |
| men 40-44 years | 96.1 | 3.9 | 0 | 99.9 | 0.1 | 0 | 98.3 | 1.7 | 0 | 92.4 | 7.2 | 0.4 | 93.9 | 5.5 | 0.6 |
| men 45-49 years | 93.9 | 5.9 | 0.2 | 98.6 | 1.3 | 0.1 | 96.0 | 3.4 | 0.6 | 88.6 | 9.7 | 1.7 | 92.2 | 6.4 | 1.4 |
| men 50-54 years | 91.8 | 7.5 | 0.7 | 97.8 | 1.9 | 0.3 | 93.0 | 6.5 | 0.5 | 82.9 | 15.1 | 2.0 | 87.9 | 11.0 | 1.1 |
| men 55-59 years | 89.9 | 9.8 | 0.3 | 96.7 | 2.7 | 0.6 | 90.4 | 8.6 | 1.0 | 76.4 | 21.9 | 1.7 | 86.6 | 12.3 | 1.1 |
| men 60-64 years | 76.1 | 23.3 | 0.6 | 96.6 | 2.4 | 1.0 | 86.6 | 11.8 | 1.6 | 66.8 | 31.0 | 2.2 | 83.5 | 15.4 | 1.1 |
| men 65-69 years | 63.7 | 34.7 | 1.6 | 89.9 | 8.6 | 1.5 | 81.6 | 16.1 | 2.3 | 55.8 | 40.3 | 3.9 | 80.6 | 17.1 | 2.3 |
| men 70-74 years | 62.2 | 36.2 | 1.6 | 90.8 | 8.1 | 1.1 | 77.5 | 19.1 | 3.4 | 53.9 | 41.8 | 4.3 | 79.7 | 18.8 | 1.5 |
| men 75-79 years | 48.9 | 47.3 | 3.8 | 80.1 | 14.7 | 4.3 | 65.0 | 27.6 | 7.4 | 39.5 | 54.9 | 5.6 | 69.5 | 27.5 | 3.0 |
| men 80-84 years | 29.3 | 66.7 | 4.0 | 66.4 | 29.7 | 3.9 | 43.4 | 50.4 | 6.2 | 25.8 | 62.5 | 11.7 | 66.3 | 29.7 | 4.0 |
| Adolescents |  |  |  |  |  |  |  |  |  |  |  |  |  |  |  |
| women 12-15 years | 99.6 | 0.2 | 0.2 | 99.8 | 0.0 | 0.2 | 99.1 | 0.7 | 0.2 | 97.3 | 2.5 | 0.2 | 97.4 | 2.4 | 0.2 |
| women 16-17 years | 100 | 0 | 0 | 100 | 0 | 0 | 100 | 0 | 0 | 99.2 | 0.8 | 0.0 | 94.9 | 5.1 | 0.0 |
| Adults |  |  |  |  |  |  |  |  |  |  |  |  |  |  |  |
| women 18-24 years | 97.7 | 1.4 | 0.9 | 97.9 | 0.9 | 1.2 | 98.0 | 0.8 | 1.2 | 95.1 | 4.6 | 0.3 | 94.7 | 4.1 | 1.2 |
| Adults by 10-yr age band |  |  |  |  |  |  |  |  |  |  |  |  |  |  |  |
| women 25-34 years | 98.3 | 1.6 | 0.1 | 99.0 | 0.7 | 0.3 | 97.9 | 1.9 | 0.3 | 94.8 | 4.9 | 0.3 | 94.2 | 4.7 | 1.1 |
| women 35-44 years | 98.2 | 1.4 | 0.4 | 99.1 | 0.6 | 0.3 | 98.2 | 1.6 | 0.2 | 91.8 | 7.6 | 0.6 | 93.2 | 5.9 | 0.9 |
| women 45-54 years | 92.8 | 7.0 | 0.2 | 98.2 | 1.5 | 0.3 | 94.9 | 4.2 | 0.9 | 84.8 | 13.8 | 1.4 | 87.5 | 11.1 | 1.4 |
| women 55-64 years | 81.0 | 18.4 | 0.6 | 95.2 | 4.3 | 0.5 | 87.1 | 12.1 | 0.8 | 69.3 | 28.4 | 2.3 | 81.2 | 16.5 | 2.3 |
| women 65-74 years | 60.7 | 38.4 | 0.9 | 90.5 | 8.9 | 0.6 | 76.0 | 22.7 | 1.3 | 46.6 | 47.7 | 5.7 | 70.1 | 26.5 | 3.4 |
| women 75-84 years | 35.8 | 60.0 | 4.2 | 76.3 | 20.8 | 2.9 | 54.8 | 40.0 | 5.2 | 26.8 | 62.5 | 10.7 | 58.0 | 37.0 | 5.0 |
| women 85+ years | 18.7 | 72.0 | 9.3 | 48.9 | 43.0 | 8.1 | 31.8 | 54.2 | 14.0 | 16.9 | 64.4 | 18.7 | 50.9 | 40.9 | 8.2 |
| Adults by 5-yr age band |  |  |  |  |  |  |  |  |  |  |  |  |  |  |  |
| women 25-29 years | 98.2 | 1.8 | 0.0 | 99.3 | 0.7 | 0.0 | 98.7 | 1.3 | 0.0 | 96.6 | 3.1 | 0.3 | 94.6 | 4.1 | 1.3 |
| women 30-34 years | 98.4 | 1.3 | 0.3 | 98.7 | 0.7 | 0.6 | 97.0 | 2.5 | 0.5 | 93.1 | 6.7 | 0.2 | 93.8 | 5.2 | 1.0 |
| women 35-39 years | 98.1 | 1.4 | 0.5 | 98.6 | 1.1 | 0.3 | 98.1 | 1.8 | 0.1 | 93.6 | 6.0 | 0.4 | 94.4 | 5.2 | 0.4 |
| women 40-44 years | 98.3 | 1.5 | 0.2 | 99.6 | 0.2 | 0.2 | 98.3 | 1.5 | 0.2 | 90.5 | 8.8 | 0.7 | 92.3 | 6.5 | 1.2 |
| women 45-49 years | 95.6 | 4.2 | 0.2 | 99.0 | 0.6 | 0.4 | 97.1 | 2.4 | 0.5 | 89.6 | 9.9 | 0.5 | 89.5 | 9.6 | 0.9 |
| women 50-54 years | 89.7 | 10.0 | 0.3 | 97.3 | 2.5 | 0.2 | 92.6 | 6.1 | 1.3 | 79.6 | 18.0 | 2.4 | 85.5 | 12.7 | 1.8 |
| women 55-59 years | 86.2 | 13.1 | 0.7 | 95.9 | 3.4 | 0.7 | 89.3 | 9.8 | 0.9 | 76.0 | 22.7 | 1.3 | 82.6 | 15.7 | 1.7 |
| women 60-64 years | 76.1 | 23.3 | 0.6 | 94.6 | 5.0 | 0.4 | 85.0 | 14.3 | 0.7 | 63.0 | 33.7 | 3.3 | 79.9 | 17.2 | 2.9 |
| women 65-69 years | 66.1 | 33.4 | 0.5 | 92.9 | 6.3 | 0.8 | 81.8 | 17.3 | 0.9 | 53.5 | 42.7 | 3.8 | 73.7 | 23.7 | 2.6 |
| women 70-74 years | 54.0 | 44.5 | 1.5 | 87.5 | 12.0 | 0.5 | 69.0 | 29.3 | 1.7 | 38.2 | 53.7 | 8.1 | 65.7 | 30.0 | 4.3 |
| women 75-79 years | 41.4 | 56.2 | 2.4 | 79.5 | 19.1 | 1.4 | 57.9 | 38.9 | 3.2 | 28.7 | 63.1 | 8.2 | 56.5 | 38.1 | 5.4 |
| women 80-84 years | 27.4 | 65.7 | 6.9 | 71.5 | 23.4 | 5.1 | 50.1 | 41.5 | 8.4 | 23.9 | 61.8 | 14.3 | 60.1 | 35.5 | 4.4 |

L1 denoting no problems, L2 denoting some/moderate problems and L3 denoting ‘extreme problems/unable to/confined to bed, yr: year

Supplementary table 3. Comparison of problems reported (based on weighted EQ-5D-3L dimensional responses) per different age bands and sex in 2022 population survey and the 2000 national health survey, expressed in % of responses, in the structure used in 2000 national health survey.

|  | EQ-5D-3L dimension | mobility | | self-care | | usual activities | | pain/discomfort | | anxiety/ depression | |
| --- | --- | --- | --- | --- | --- | --- | --- | --- | --- | --- | --- |
|  | level | L1 | L2+L3 | L1 | L2+L3 | L1 | L2+L3 | L1 | L2+L3 | L1 | L2+L3 |
| 2000 survey | men  18-34 | 97.5  [96.3-98.3] | 2.5  [1.7-3.7] | 99.1  [98.1-99.9] | 0.9  [0.4-1.9] | 98.2  [96.8-99.0] | 1.8  [1.0-3.2] | 85.2  [82.2-87.8] | 14.8  [12.2-17.8] | 85.0  [82.5-87.2] | 15.0  [12.8-17.5] |
|  | men  35-64 | 83.0  [79.5-86.0] | 17.0  [14.0-20.5] | 95.2  [93.5-96.5] | 4.8  [3.5-6.5] | 86.8  [84.6-88.7] | 13.2  [11.3-15.4] | 64.2  [59.3-68.8] | 35.8  [31.2-40.7] | 70.0  [66.4-73.4] | 30.0  [26.6-33.6] |
|  | men  65+ | 61.1  [55.9-66.0] | 38.9  [34.0-44.1] | 82.9  [78.6-86.4] | 17.1  [13.6-31.4] | 69.1  [64.1-73.7] | 30.9  [26.3-35.9] | 44.1  [39.2-49.1] | 55.9  [50.9-60.8] | 59.5  [54.6-64.2] | 40.5  [35.8-45.4] |
|  | women  18-34 | 96.8  [95.2-97.8] | 3.2  [2.2-4.8] | 99.7  [99.0-99.9] | 0.3  [0.1-1.0] | 97.2  [96.0-98.1] | 2.8  [1.9-4.0] | 81.8  [78.0-85.0] | 18.2  [15.0-22.0] | 73.9  [70.4-77.1] | 26.1  [22.9-29.6] |
|  | women  35-64 | 79.3  [76.7-81.6] | 20.7  [18.4-23.3] | 94.7  [93.3-95.8] | 5.3  [4.2-6.7] | 84.4  [82.1-86.5] | 15.6  [13.5-17.9] | 52.0  [48.2-55.8] | 48.0  [44.2-51.8] | 55.5  [53.0-57.9] | 44.5  [42.1-47.0] |
|  | women  65+ | 50.6  [46.1-55.1] | 49.4  [44.9-53.9] | 81.0  [77.0-84.4] | 19.0  [15.6-23.0] | 64.5  [60.3-68.5] | 35.5  [31.5-39.7] | 31.6  [28.4-35.0] | 68.4  [65.0-71.6] | 45.7  [42.0-49.5] | 54.3  [50.5-58.0] |
| 2022 survey | men  18-34 | 98.1  [97.1-98.8] | 1.9  [1.2-2.9] | 98.7  [97.7-99.3] | 1.3  [0.7-2.3] | 97.8  [96.5-98.6] | 2.2  [1.4-3.5] | 95.1  [93.5-96.3] | 4.9  [3.7-6.5] | 94.0  [91.6-95.7] | 6.0  [4.3-8.3] |
|  | men  35-64 | 91.6  [90.4-92.8] | 8.4  [7.2-9.6] | 98.3  [97.8-98.8] | 1.7  [1.2-2.2] | 94.4  [93.2-95.4] | 5.6  [4.6-6.8] | 84.5  [82.6-86.1] | 15.5  [13.9-17.4] | 90.2  [88.8-91.5] | 9.8  [8.5-11.2] |
|  | men  65+ | 54.5  [51.1-57.8] | 45.5  [42.2-48.9] | 83.9  [81.2-86.2] | 16.1  [13.8-18.8] | 70.6  [67.4-73.6] | 29.4  [26.4-32.6] | 47.3  [43.9-50.7] | 52.7  [49.3-56.1] | 75.0  [72.0-77.8] | 25.0  [22.2-28.0] |
|  | women  18-34 | 98.1  [96.8-98.8] | 1.9  [1.2-3.2] | 98.6  [97.5-99.2] | 1.4  [0.8-2.5] | 97.9  [96.7-98.7] | 2.1  [1.3-3.3] | 94.9  [93.3-96.2] | 5.1  [3.8-6.7] | 94.4  [92.5-95.8] | 5.6  [4.2-7.5] |
|  | women  35-64 | 91.1  [89.8-92.2] | 8.9  [7.8-10.2] | 97.6  [96.9-98.2] | 2.4  [1.8-3.1] | 93.7  [92.6-94.6] | 6.3  [5.4-7.5] | 82.5  [80.6-84.2] | 17.5  [15.7-19.4] | 87.6  [85.9-89.0] | 12.4  [11.0-14.1] |
|  | women  65+ | 48.5  [45.8-51.2] | 51.5  [48.8-54.2] | 82.1  [79.7-84.2] | 17.9  [15.8-20.3] | 64.9  [62.2-67.5] | 35.1  [32.5-37.8] | 37.2  [34.4-40.1] | 62.8  [59.9-65.6] | 64.3  [61.3-67.2] | 35.7  [32.8-38.7] |

L1 denoting no problems, L2 denoting some/moderate problems and L3 denoting ‘extreme problems/unable to/confined to bed

Supplementary table 4. Comparison of weighted EQ-5D-3L index values (using the UK value set by Dolan et al.) within the 2022 population survey and the 2000 national health survey per 10-year age bands and sex

|  | Mean | SE | 95% CI | Mean | SE |
| --- | --- | --- | --- | --- | --- |
| Age band | 2022@UK value set | | | 2000@UK value set | |
| men 18-24 years | 0.973 | 0.007 | (0.959; 0.988) | 0.951 | 0.006 |
| men 25-34 years | 0.968 | 0.005 | (0.959; 0.978) | 0.936 | 0.006 |
| men 35-44 years | 0.972 | 0.004 | (0.965; 0.979) | 0.895 | 0.009 |
| men 45-54 years | 0.936 | 0.007 | (0.923; 0.949) | 0.834 | 0.012 |
| men 55-64 years | 0.887 | 0.008 | (0.872; 0.902) | 0.808 | 0.014 |
| men 65-74 years | 0.802 | 0.010 | (0.782; 0.823) | 0.768 | 0.018 |
| men 75-84 years | 0.675 | 0.017 | (0.641; 0.709) | 0.671 | 0.029 |
| men 85+ years | 0.540 | 0.046 | (0.450; 0.631) | 0.677 | 0.061 |
| women 18-24 years | 0.966 | 0.010 | (0.946; 0.986) | 0.932 | 0.007 |
| women 25-34 years | 0.971 | 0.004 | (0.963; 0.979) | 0.898 | 0.009 |
| women 35-44 years | 0.965 | 0.004 | (0.956; 0.974) | 0.871 | 0.009 |
| women 45-54 years | 0.930 | 0.006 | (0.919; 0.942) | 0.781 | 0.012 |
| women 55-64 years | 0.869 | 0.008 | (0.854; 0.884) | 0.728 | 0.012 |
| women 65-74 years | 0.769 | 0.009 | (0.751; 0.787) | 0.682 | 0.016 |
| women 75-84 years | 0.633 | 0.016 | (0.601; 0.666) | 0.615 | 0.024 |
| women 85+ years | 0.475 | 0.031 | (0.415; 0.536) | 0.609 | 0.048 |

SE: standard error; CI: confidence interval; UK: United Kingdom

Supplementary table 5. Comparison of weighted EQ VAS within the 2022 population survey and the 2000 national health survey

|  | 2000 | 2022 |  |
| --- | --- | --- | --- |
| Age band, men | Mean | Mean | 95% CI |
| 18-24 | 84.2 | 92.4 | 91.3-93.6 |
| 25-34 | 81.7 | 89.4 | 88.2-90.5 |
| 35-44 | 76.1 | 89.0 | 88.0-90.0 |
| 45-54 | 70.4 | 84.2 | 83.1-85.4 |
| 55-64 | 66.2 | 78.9 | 77.4-80.3 |
| 65-74 | 62.9 | 71.9 | 70.3-73.4 |
| 75-84 | 55.4 | 63.2 | 60.7-65.6 |
| 85+ | 55.1 | 56.8 | 52.2-61.5 |
| Age band, women | Mean | Mean | 95% CI |
| 18-24 | 82.6 | 92.4 | 91.1-93.7 |
| 25-34 | 80.1 | 90.4 | 89.4-91.4 |
| 35-44 | 75 | 87.8 | 86.7-89.0 |
| 45-54 | 68.3 | 83.9 | 82.8-85.0 |
| 55-64 | 62.4 | 77.3 | 76.0-78.5 |
| 65-74 | 57.2 | 70.7 | 69.4-71.9 |
| 75-84 | 53.2 | 62.7 | 60.7-64.6 |
| 85+ | 54.3 | 55.5 | 52.3-58.7 |

CI: confidence interval
